# The Cardiac Output–Cerebral Blood Flow Relationship Is Abnormal in Most Myalgic Encephalomyelitis/Chronic Fatigue Syndrome Patients with a Normal Heart Rate and Blood Pressure Response During a Tilt Test

**DOI:** 10.3390/healthcare12242566

**Published:** 2024-12-20

**Authors:** C (Linda) M. C. van Campen, Freek W. A. Verheugt, Peter C. Rowe, Frans C. Visser

**Affiliations:** 1 Stichting Cardio Zorg, Kraayveld 5, 1171 JE Badhoevedorp, The Netherlands; fransvisser@stichtingcardiozorg.nl; 2 Department of Cardiology, Onze Lieve Vrouwe Gasthuis (OLVG), 1091 AC Amsterdam, The Netherlands; 3 Department of Paediatrics, John Hopkins University School of Medicine, Baltimore, MD 21205, USA; prowe@jhmi.edu

**Keywords:** stroke volume, cardiac output, cerebral blood flow, tilt table test, orthostatic intolerance, chronic fatigue syndrome (CFS), myalgic encephalomyelitis (ME), healthy controls

## Abstract

Introduction: Orthostatic intolerance is highly prevalent in patients with myalgic encephalomyelitis/chronic fatigue syndrome (ME/CFS) and is caused by an abnormal reduction in cerebral blood flow (CBF). In healthy controls (HCs), the regulation of CBF is complex and cardiac output (CO) is an important determinant of CBF: a review showed that a 30% reduction in CO results in a 10% reduction in CBF. In previous and separate ME/CFS studies, we showed that CO and CBF decreased to a similar extent during tilt testing. The aim of the study: to test the relationship between CBF and CO, which seems to be abnormal in ME/CFS patients and is different from that in HCs. Methods: In this retrospective study we analyzed this relationship in a large group of patients. To compare the patient data with those of HCs, we focused on patients with a normal heart rate (HR) and blood pressure (BP) response to upright tilt. Also, the influence of clinical data was analyzed. A total of 534 ME/CFS patients and 49 HCs underwent tilt testing with measurements of HR, BP, CBF, CO, and end-tidal PCO_2_. To measure CBF, extracranial Doppler flow velocity and vessel diameters were obtained using a GE echo system. The same device was used to measure suprasternal aortic flow velocities. End-tidal PCO_2_ was recorded using a Nonin Lifesense device. Results: In 46 (9%) patients, CO and CBF changes were in the normal range for HCs, and in 488 (91%) an abnormal CO and CBF reduction was found. In patients with abnormal CO and CBF reductions, the slope of the regression line of CO versus CBF reduction was almost 1. The multiple regression analysis of the latter group showed that the CO reduction for the most part predicted the CBF reduction, with a limited role for the P_ET_CO_2_ reduction. Conclusions: Two different patient groups with a normal HR and BP response during the tilt were identified: those with a CO and CBF in the normal range for HCs and those with an abnormal CO and CBF reduction during the tilt (91% of patients). In the latter group of patients, an almost 1:1 relationship between the CO and CBF reduction suggests the absence of compensatory vasodilation in the cerebral vasculature. This might indicate endothelial dysfunction in most ME/CFS patients and may have clinical and therapeutic implications.

## 1. Introduction

Orthostatic intolerance is a well-described phenomenon in patients with myalgic encephalomyelitis/chronic fatigue syndrome (ME/CFS) [1,2,3]. The symptoms of orthostatic intolerance—provoked by sitting, standing, upright activity, or tilt testing—are caused by an abnormal reduction in cerebral blood flow (CBF). The complex and only partially understood mechanisms of cerebral flow regulation have been studied in healthy controls and patients with a variety of diseases. The mechanisms involved include cerebral perfusion pressure, PO_2_ and PCO_2_, flow–metabolism coupling, innervation of cerebral vessels, and blood viscosity [4]. Also, age and gender influence CBF. In ME/CFS patients, an abnormally decreased venous return due to orthostatic stress [5,6], differences in blood volume [7,8], leg venous distensibility [9,10], muscle blood pump characteristics [11], deconditioning [12], sympathetic drive [13], hemodynamic abnormality during a tilt test [2], chronotropic incompetence [14,15], neuroinflammation [16], the autoimmunity of the nervous system [17], endothelial dysfunction [18], disease severity [19], and microclots [20] may play a role.

Another important determinant of CBF is the cardiac output (CO). In a review, Meng et al. described the relationship between changes in CO and changes in CBF in healthy controls (HCs) during a variety of interventions, aiming to reduce or increase the CO [21]. Summarizing the results of five studies with CO reductions in HCs, the authors found that a reduction of 30% in CO resulted in a 10% reduction in CBF. On the other hand, Castle-Kirszbaum et al. found in their systematic review that the “current literature is insufficiently robust to confirm an independent relationship between CO and CBF” [22].

We have demonstrated in different studies of ME/CFS patients that (1) there is a significantly larger reduction in cardiac index (CI) during a tilt test compared to HCs [6,23], and (2) there is a significantly larger reduction in CBF during the tilt test compared to HCs [2,24,25]. Importantly, CI and CBF improved in these patients while wearing compression stockings, compared to the group without compression stockings [26], suggesting a causal role for the CO reduction on the CBF reduction. Theoretically, the larger CBF reduction in ME/CFS patients follows the larger CO reduction, like the changes in HCs (30% CO reduction/10% CBF reduction), or, alternatively, a larger than expected CBF reduction is present. Based on previous findings of a mean abnormal CBF and a mean abnormal CO reduction in ME/CFS patients, compared to HCs, we hypothesized that the relationship between CBF and CO—as part of the cerebral flow regulation [21,27]—is abnormal in ME/CFS patients and is different from that in HCs during orthostatic stress like a tilt test. The aim of the study was to test this hypothesis with analysis from the large available clinical database.

## 2. Materials and Methods

We reviewed the medical records of all ME/CFS patients who visited the out-patient clinic from October 2012 to May 2022 and who underwent a tilt test. From the first visit, we determined whether participants satisfied the criteria for ME and for CFS [28,29], taking the exclusion criteria into account. No other illnesses were present that explained the symptomatology. Patients were selected for analysis when, from the tilt test, Doppler data for both CO and CBF were available, both in the supine position and in the upright phase of the test. No drugs influencing HR or BP were used at the time of the tilt testing. For comparison, healthy controls were studied. Healthy controls were recruited from three sources: (a) announcements on ME/CFS patient advocacy websites, (b) posters in the medical clinic’s office building, and (c) healthy acquaintances of the ME/CFS participants. Subjects referred for syncope analysis or for other cardiologic diseases at our clinic were not considered as healthy controls. In the present study, we studied both ME/CFS patients and HCs. As the HCs studied in the present study showed a normal heart rate (HR) and blood pressure (BP) response during the tilt test, we only analyzed the ME/CFS patients with a normal HR and BP response during the tilt and excluded those with postural orthostatic tachycardia syndrome (POTS), orthostatic hypotension (OH), or a (near)-syncope.

Disease severity in patients was scored according to the international consensus criteria (ICC), with severity scored as mild, moderate, severe, and very severe [29]. Very severe patients (bedridden patients) were not studied here because they were not able to undergo a tilt test.

The study was carried out in accordance with the Declaration of Helsinki. All ME/CFS participants and healthy controls gave informed, written consent. The study was approved by the medical ethics committee of the Slotervaart Hospital, Amsterdam, for healthy controls P1450 and for ME/CFS patients P1736.

### 2.1. Tilt Test Protocol

Measurements were performed as described previously [2,30]. Briefly, all participants were positioned for 20 min in a supine position before being tilted head-up to 70 degrees. Tilt duration was maximally 30 min, but in patients tilt duration was shorter and determined by the patient’s wellbeing and symptomatology; with increasing symptoms patients were tilted for a shorter period of time to avoid syncope and to avoid possible post-exertional malaise.

HR, systolic, and diastolic blood pressures (SBP and DBP) were continuously recorded by finger plethysmography [31,32]. After the test, HR and BP were extracted from the device and imported into an Excel spreadsheet. The changes in HR and BP during tilt testing were classified according to the consensus statement [33,34,35]: normal HR and BP response (normal HR-BP response), classic orthostatic hypotension (cOH), delayed orthostatic hypotension (dOH), postural orthostatic tachycardia syndrome (POTS), and (near-) syncope. For comparison with HCs, in the present study we only analyzed patients with a normal HR-BP response where blood pressures were within normal limits.

### 2.2. Extracranial Doppler: Cerebral Blood Flow Measurements

To measure CBF, we used extracranial Doppler due to the inherent limitations of transcranial Doppler [22]. Measurements were performed as described previously [2,30]. Internal carotid artery and vertebral artery Doppler flow velocity frames were acquired by one operator (FCV), using a Vivid-I system (GE Healthcare, Hoevelaken, The Netherlands) equipped with a 6–13 MHz linear transducer. As described previously [30], flow data of the ICA were obtained 1.0–1.5 cm distal to the carotid bifurcation and of the VA at the C3–C5 level. Care was taken that the insonation angle was less than 60 degrees, that the sample volume was positioned in the center of the vessel, and that it covered the width of the vessel. High-resolution B-mode images, color Doppler images, and the Doppler velocity spectrum (pulsed-wave mode) were recorded in one frame. The order of imaging was fixed: left ICA, left VA, right ICA, and right VA. At least 2 consecutive series of 6 s per artery were recorded. Frames were recorded in the supine position, before the onset of the tilt period, and while upright once or twice. When two sets of cerebral flow acquisitions were available, only the last set was analyzed. The blood flow of the internal carotid and vertebral arteries was calculated offline by one investigator (CMCvC). Blood flow in each vessel was calculated from the mean blood flow velocities times the vessel cross-sectional area and expressed in mL/minute. Surface area was calculated as proposed by Sato et al. (2012): the peak systolic and end-diastolic diameters were measured, and the mean diameter was calculated as mean diameter = (peak systolic diameter × 1/3) + (end-diastolic diameter × 2/3) [36]. Flow in the individual arteries was calculated in 3–6 cardiac cycles and data were averaged. Total CBF was calculated by adding the flow of the four arteries. The difference in total upright CBF minus total supine CBF divided by total supine CBF multiplied by 100% was used as the percentage of the reduction in CBF. As described in our first manuscript describing cerebral blood flow abnormalities in ME/CFS patients compared to healthy controls, the end-tilt % cerebral blood flow decrease in controls was 7 (3)%. Assuming a lower limit of normal of 2 SD below the mean for healthy controls, the lower limit of a normal % cerebral blood flow decrease during the tilt was 13% [2].

### 2.3. End-Tidal CO_2_ Pressure Measurements

Moreover, apart from clinical data, HR, and BP, we also analyzed the influence of end-tidal PCO_2_ (P_ET_CO_2_), which has a powerful influence on CBF [4]. End-tidal CO_2_ pressures (P_ET_CO_2_) (in mmHg) were continuously measured with the Nonin Lifesense device. End-tilt pressures below 30 mmHg were considered abnormal [37].

### 2.4. Doppler Echocardiographic Measurements

Time–velocity integral (VTI) frames were obtained in the resting supine position and at end of the tilt phase, immediately after the cerebral flow acquisitions. The aortic VTI was measured using a continuous-wave Doppler pencil probe connected to a Vivid I machine (GE, Hoevelaken, The Netherlands) with the transducer positioned in the suprasternal notch. A maximal Doppler signal was assumed to be the optimal flow alignment. At least 2 frames of 6 s were obtained. Echo-Doppler recordings were stored digitally. The VTI was measured offline by manual tracing of at least 6 cardiac cycles, using the GE EchoPac post-processing software by one operator (CMCvC). The outflow tract diameter was manually drawn just below the valve insertion in the parasternal long-axis view of a previously made echocardiogram and the cross-sectional area was calculated. As the outflow tract is not circular but ellipsoid, we used the data of Maes et al. [38] to correct for the overestimation by the circular shape of the ellipsoid ventricular outflow tract calculation. In their study, the overestimation of the outflow tract area, using the circular calculation by transthoracic echocardiography, was 24.5%. Therefore, we reduced the outflow tract area by 25%. Stroke volume (SV) was calculated from the aortic VTI, multiplied by the corrected aortic valve area, and expressed in mL. The SVs of the separate cycles were averaged. CO was calculated by the formula: SV times HR and expressed in L/min. The upright (end-of-study) CO minus the supine CO divided by the supine CO multiplied by 100% was used as the percentage of reduction in CO. In line with the method used regarding describing cerebral blood flow abnormalities in ME/CFS patients compared to healthy controls, the end-tilt % cardiac output decrease in controls was 10 (5)%. Assuming a lower limit of 2 SD below the mean for healthy controls, the lower limit of a normal % cardiac output decrease during the tilt was 20% (data not shown; in healthy controls, distribution was normal, in ME/CFS patients it was not).

### 2.5. Statistical Analysis

Data were analyzed using the statistical package of IBM SPSS, version 29.0.00.0. All continuous data were tested for normal distribution using visual inspection of the Q-Q plots and presented as mean and standard deviation (SD) or as median with the interquartile range (IQR) where appropriate. Nominal data were compared using the Chi-square test (gender and disease severity, 3 × 2 and 3 × 3 tables). Group differences were explored using Welch ANOVA, by the Mann–Whitney U test in case of the comparison of two groups or by the Kruskal–Wallis test for the comparison of three or more groups. Post hoc tests were performed using the Tukey or Dunn test. Multiple regression analysis was performed according to the guidelines of Laerd Statistics [39]. The slopes of the regression line of the three groups were compared using the statistical package GraphPad Prism v9.5.1, GraphPad Software, San Diego, CA, USA. Due to the large number of comparisons, to reduce type I errors, we chose a conservative *p*-value of <0.01 to be statistically significant.

## 3. Results

From our database, we selected ME/CFS patients who had visited our clinic between Oktober 2012 and May 2022, who fulfilled the criteria for both ME and CFS [28,29], and who had a tilt test because of the suspicion of orthostatic intolerance (n = 1135). In all patients, CBF measurements as well as suprasternal-derived SV were available. From this group, we selected patients with a normal HR-BP response (n = 664). Patients younger than 18 years were excluded leaving 629 patients. Also, patients with a BMI > 40 were excluded, leaving 612 patients. Patients with CBF or SV measurements of insufficient quality were excluded, as were patients with missing data for CBF and SV, leaving 585 patients. Finally, 51 patients using HR- and BP-lowering drugs or lung medication containing sympathomimetics were excluded. This left 534 patients to be analyzed. For comparison with the patients, 49 healthy controls (HCs) with a normal HR-BP response, and with a complete set of CO and CBF measurements were also analyzed. A flow diagram clarifying this selection is shown as Supplementary Figure S1 in the Supplementary Materials.

**Figure 1:**
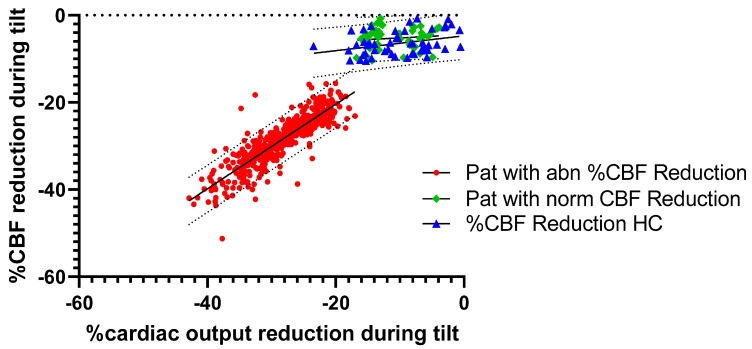
Percentage cerebral blood flow reduction vs. percentage cardiac output reduction during the tilt test in ME/CFS patients and healthy controls. Legend: abn: abnormal; %CBF Red: percentage reduction in cerebral blood flow during the tilt; HC: healthy controls; Pat: patients; ME/CFS: myalgic encephalomyelitis/chronic fatigue syndrome; norm: normal.

Based on the distribution of the %CO reduction versus the %CBF reduction (see Figure 1) we decided to separate patients into a group where the %CBF reduction was in the normal range of the healthy controls, and a group of patients with a %CBF reduction below the lower limit of normal for the healthy controls (a cut-off value of %CBF reduction of −15%). Table 1 shows the baseline characteristics of the three groups.

Four hundred and eighty-eight patients had a larger than normal %CBF reduction, while forty-six showed a %CBF reduction in the normal range of the HCs. There were significantly more males in the patient group with a %CBF reduction in the normal range. Patients with an abnormal %CBF reduction were more severely affected by the disease, with a higher percentage of moderate and severe disease than patients with a %CBF reduction in the normal range. All other baseline characteristics were similar between the three groups.

Table 2 shows the hemodynamic data of the three groups in the supine position and at the end of the tilt phase. Supine and end-tilt HR were significantly higher in the patients with an abnormal %CBF reduction compared to HCs. Supine CO was higher in patients with an abnormal %CBF reduction during the tilt compared to HCs. End-tilt CO was lowest in patients with an abnormal %CBF reduction compared to patients with a normal %CBF reduction and compared to HCs. Accordingly, the %CO reduction at end-tilt was significantly larger in the patient group with an abnormal %CBF reduction. By definition, the end-tilt CBF was lower and the %CBF reduction larger in the patient group with the abnormal %CBF reduction compared to the other patient group and HCs. The P_ET_CO_2_ reduction was significantly larger in patients with an abnormal %CBF reduction than those with an %CBF reduction in the normal range of HCs and compared to the P_ET_CO_2_ reduction in HCs. Also, the %patients with an P_ET_CO_2_ < 30 mmHg was larger in patients with an abnormal %CBF reduction. End-tilt DBPs were higher in both patient groups compared to HCs but reached significance only in the patient group with an abnormal %CBF reduction. End-tilt MAP was significantly higher in both patient groups compared to HCs, but only reached significance in patients with an abnormal %CBF reduction. Tilt duration was significantly shorter in patients with an abnormal %CBF reduction compared to patients with a %CBF reduction in the normal range and compared to HCs.

Frame acquisition for CBF measurements lasted 3.3 (1.6) minutes without differences between groups (data not shown). The acquisition of VTI measurements lasted 0.7 (0.3) minutes without differences between groups (data not shown).

Figure 1 shows the relationship between the %CO reduction and %CBF reduction in the three groups. In patients with an abnormal %CBF reduction, the relationship was highly significant: %CBF reduction = 0.971 × %CO reduction − 1.027; R^2^ = 0.762; *p* < 0.001. In patients with a normal %CBF reduction, the slope was not significantly different from zero: %CBF reduction = 0.076 × %CO reduction − 4.420; R^2^ = 0.014; *p* = 0.438. In HCs, the slope was marginally significant: %CBF reduction = 0.171 × %CO reduction − 4.708; R^2^ = 0.114; *p* = 0.018. The slope of the relationship between the %CO and %CBF reduction was significantly different between patients with an abnormal %CBF reduction and HCs (*p* < 0.001). The slopes of patients with a normal %CBF reduction and of HCs were not significantly different.

To determine whether clinical and other hemodynamic variables were associated with the %CBF reduction during the tilt, we analyzed the relationship between %CBF reduction in the patient group with an abnormal %CBF reduction. Patients with a normal %CBF reduction were not analyzed as the regression line was not different from zero. In this subgroup of patients with an abnormal %CBF reduction, 440/488 (90%) had adequate P_ET_CO_2_ measurements (43% of these 440 patients had a P_ET_CO_2_ below 30 mmHg). The %CBF reduction was related to gender, age, disease duration and severity, length, weight, the heart rate increase, the %CO reduction, P_ET_CO_2_ reduction, patients with a P_ET_CO_2_ < 30 mmHg, the %MAP increase, and tilt duration. Table 3 shows the results of the univariate analysis: increasing disease severity was associated with a larger %CBF reduction, a larger %CO reduction, a larger P_ET_CO_2_ reduction, and an end-tilt P_ET_CO_2_ < 30 mmHg resulting in significantly larger %CBF reductions. Other variables showed no significant relationships. For the multivariate analysis, variables were included that showed a *p* value < 0.1 in the univariate analysis.

Table 4 shows that the %CO reduction and tilt duration significantly predicted the %CBF reduction. The P_ET_CO_2_ reduction showed a marginal contribution (*p* = 0.011). All other variables did not significantly contribute to the model. The model explained 78% of the variation.

## 4. Discussion

The main findings in the present study are that the %CBF and %CO reductions in patients are significantly larger those of HCs. Second, in a large part of ME/CFS patients (those with an abnormal %CBF reduction: in 91%) the relationship between %CO and %CBF is significantly different from the relationship in HCs and in patients with a %CBF reduction in the normal range of HCs: in the former patient group (with an abnormal %CBF reduction), the relationship between %CBF and %CO reduction was almost 1:1, indicating the absence of compensatory vasodilation of the cerebral vessels. This may indicate endothelial dysfunction. Third, the multiple regression analysis showed that the %CO reduction was the major determinant of the %CBF reduction with an unstandardized B (slope) of 0.959. The absolute P_ET_CO_2_ reduction was marginally significant (*p* = 0.011), while tilt duration had a small negative but significant effect on %CBF reduction (unstandardized B: −0.063; *p* = 0.003), with only little influence of P_ET_CO_2_ differences as has been suggested in previous studies [37]. A previous study already showed a less important contribution of P_ET_CO_2_ difference, and this was confirmed in the present study [23]. In the present study, two different patterns in patients were observed: first, a limited number of patients (9%) had a %CBF reduction (−5%) and a %CO reduction (−13%) during the tilt. These values were in the range of HCs, without significant differences between these patients and HCs (Table 2). In contrast, most patients (91%) showed a large and abnormal %CBF reduction during the tilt, with a mean % reduction of −29% and a %CO reduction of −28%.

The data of the HCs in the present study were compared with those in the review by Meng [21]. The %CBF reduction in the five reviewed studies varied between −4 and −19%, the %CO reduction between −18 and −44, while in our study the %CBF reduction was −6% and the %CO −10%. However, there are differences in techniques used for both the CBF (transcranial Doppler vs. extracranial Doppler in our study) and the CO measurements (inert gas/acetylene rebreathing, finger plethysmography, and impedance cardiography vs. suprasternal Doppler in our study). Also, the orthostatic stressor was different between our study (tilt test) and the previous ones (mainly lower-body negative pressure (LBNP) and standing up). These differences in measurement techniques and stressors may account for the differences in the obtained CO and CBF data. For example, in a direct comparison study between LBNP and a tilt test, the CBF reduction by LBNP in HCs was significantly less than during tilt testing, which the authors attributed to differences in P_ET_CO_2_ [40]. Also, we demonstrated that CO by finger plethysmography underestimates the CO changes during the tilt compared to suprasternal Doppler [41].

When comparing ME/CFS patients with a %CBF reduction in the normal range of HCs and patients with an abnormal %CBF reduction, patients with a %CBF reduction in the normal range of HCs had less severe ME/CFS and were more likely to be male than patients with abnormal CBF and CO reductions. We have recently shown that disease severity, classified according to the ICC [29], was related to the %CBF reduction: severe patients had a larger %CBF reduction during the tilt test than patients with a mild or moderate severity of the disease [25]. The association between increasing symptom severity and a larger %CBF reduction is strengthened by this study, showing that patients with worsening symptoms over time (including those initially with a %CBF reduction in the normal range) had a larger %CBF reduction during the second tilt test [25]. Furthermore, Streeten et al. and our group showed that the use of military anti-shock trousers/compression stockings improved both OI and ME/CFS symptomatology and CBF abnormalities in ME/CFS patients [8,26,42].

Although there is an association between ME/CFS disease severity and the %CBF reduction, there is no evidence that there is a cause–effect relationship. This is evident from the patient group without an abnormal CBF reduction, but with symptoms varying between mild and severe disease.

The observation that males have less severe ME/CFS has been observed previously, reflected by fewer symptoms and a higher physical functioning scale than women [43,44,45]. The mechanism of milder symptomatology in men is unknown, as is the observation of a lower prevalence of the disease in men.

The largest group of ME/CFS patients are those with an abnormal %CBF reduction during the tilt. As shown in Figure 1, the %CBF reduction parallels the %CO reduction with a slope between the %CBF and %CO reduction that is near to 1. In the subgroup analysis of patients for whom P_ET_CO_2_ data were also available (n = 440), clinical variables like disease severity and an abnormal P_ET_CO_2_ (<30 mmHg) were associated with the abnormal %CBF reduction (see Table 3). However, the multiple regression analysis showed that only the %CO reduction was significantly associated with the %CBF reduction. The other variables did not contribute significantly.

One may divide mechanisms that regulate CBF into four distinct components or adaptive responses: autoregulation; chemoregulation, also called vascular reactivity; neuronal regulation, including the neurovascular coupling and the effects of autonomic and sensory nerves on the extraparenchymal segments of the cerebral vasculature; and endothelium-dependent regulation [27,46]. Autoregulation refers to how CBF responds to changes in blood pressure. In the present study, there are minimal changes in DBP, and MAP, suggesting that autoregulation plays a minor role here. Second, chemoregulation by CO_2_ (vascular reactivity) is probably normal in these patients as in the univariate analysis the %CBF reduction was related to the P_ET_CO_2_ reduction and the mean P_ET_CO_2_ reduction was 8 mmHg and the %CBF reduction was −29%. This relationship is in line with a number of studies of HCs, where a 1 mmHg reduction in P_ET_CO_2_ was related to a 3–4% reduction in CBF (see review by Hoiland et al. [4]). Nevertheless, in the multivariate analysis, the P_ET_CO_2_ reduction was marginally related to the %CBF reduction due to our choice to set the significance level to 0.01, to reduce false-positive findings. Third, neurovascular coupling describes the close temporal and regional linkage between neural activity and CBF responses, where an increase in neuronal activity leads to an increase in CBF by the dilatation of upstream pial arteries and nearby arterioles and capillaries [47,48]. In humans, neurovascular coupling can be studied, e.g., using a visual stimulation with an eye-open and eye-closed protocol. Although in our protocol leg muscle tone may increase during standing, thereby increasing regional CBF, the large reduction in CO and CO_2_, together with the assessment of global CBF prevents assessment of neurovascular coupling. Fourth, the cerebrovascular endothelium exerts a profound influence on cerebral vessels and cerebral blood flow via smooth muscle cell dilators and constrictors, like NO and endothelin-1 [49,50]. Even under conditions of high orthostatic stress, cerebral flow regulation in HCs is preserved, as a study by Brown et al. showed that a mean %CO reduction of −44% resulted in a %CBF reduction of −19% [51]. Given the observation that the %CBF reduction is related to the %CO reduction in almost a 1:1 relationship in over 90% of the ME/CFS patients with a normal HR-BP response, it is most likely that endothelial dysfunction of the cerebral vasculature plays a dominant role in abnormal cerebral flow regulation during orthostatic stress. As outlined in the introduction, the mechanisms of cerebral flow regulation in HCs are complex, but in ME/CFS patients additional factors that may disturb cerebral flow regulation, like abnormal venous return, blood volume changes, venous distensibility, deconditioning, chronotropic incompetence, neuroinflammation, autoimmunity, and microclots, can also play a role. Endothelial dysfunction in the brain has been described in a variety of cerebral diseases like Alzheimer disease [52], cerebral small-vessel disease [50], in multiple sclerosis [53], and possibly in Parkinson’s disease [54]. In ME/CFS patients, a number of studies have shown endothelial dysfunction using flow-mediated vasodilation/post-occlusive hyperemia [18,55,56,57,58]. Our study, specifically targeting the cerebral vasculature, adds to evidence that endothelial dysfunction of the brain may be present in the majority of patients.

Figure 1 shows that there is a separation between patients with and without an abnormal %CBF reduction with a cut-off value of the %CBF reduction set at around −15%. This observation is strengthened by our previous study where patients with worsening symptoms showed a substantial further reduction in %CBF [25]. Twenty-five of these patients with worsening symptoms (n = 71) initially had a %CBF reduction in the normal range of HCs. In this subgroup, the initial %CBF reduction was −6% (SD 4%) but changed to −25% (SD 5%), a value well beyond the cut-off value of −15%. Possibly, one of the above-mentioned mechanisms in ME/CFS may become operational (venous return, hypovolemia, deconditioning, sympathetic activation, chronotropic incompetence, neuro-inflammation, auto-immunity, endothelial dysfunction, and micro clots), but this needs to be studied further.

Finally, the multiple regression shows that the %CBF change was inversely correlated with the tilt duration. Although the slope of this regression line was significantly different from zero, the slope itself was −0.066, suggesting a minimal change from a short tilt to a long tilt duration. This has also been observed in an earlier study [2].

### Limitations

We acknowledge that the present study is retrospective and referral bias by the general practitioner may have played a role, selectively referring patients with orthostatic symptoms. Although this might have affected the generalizability of our CBF measurements to the entire population of individuals with ME/CFS, it would not have interfered with the validity of the observed relationships between CO and CBF. Our study did not enroll those who were bedbound, and we elected not to expose those with more severe functional impairments to tilt testing. Moreover, we only selected those patients with a normal HR and BP response during tilting. The same analysis should be performed in patients with POTS and orthostatic hypotension. Individuals with ME/CFS have been reported to have variable function from day to day and week to week. Future studies can evaluate whether the CBF and CO measurements differ on “good” versus “bad” days. Our focus was on the prevalence of reductions in CBF and CO and therefore the mechanisms of CBF and CO changes and regional cerebral blood flow differences were beyond the scope of this study. We discussed four potential mechanisms involved to some extent. In this retrospective study on clinical information, we were not able to distinguish between these mechanisms. These topics would be important to investigate further.

Finally, the use of extracranial Doppler flow to measure cerebral blood flow must be replicated by others and in different patient groups. It is unclear how much the orthostatic intolerance of ME/CFS patients differs from other forms of circulatory dysfunction. As with transcranial Doppler (TCD), only velocities are measured in the arteria cerebri media and no difference in measurement position or change in diameter due to orthostatic stress factors can be taken into account with this methodology; extracranial Doppler seems to be more accurate [59,60]

## 5. Conclusions

In ME/CFS patients undergoing tilt testing because of the suspicion of orthostatic intolerance, with measurements of both the CO and the CBF, two different patterns were observed: limited number of patients (9%) had a %CBF reduction in the range of HCs. This group of patients had a milder severity of the disease compared to the group of patients with an abnormal %CBF reduction and contained more males. The largest group of patients (91%) were those with an abnormal %CBF reduction. In the latter group, multiple regression analysis with clinical, hemodynamic, and P_ET_CO_2_ data showed that the %CO reduction robustly predicted the %CBF reduction, with minor contributions of the P_ET_CO_2_ and the tilt duration. These data suggest that circulatory improvement by increased water and salt intake, compression garments, and medications targeted to improve CO are the primary targets to improve orthostatic intolerance. This needs to be prospectively assessed in randomized, placebo-controlled trials.

## Figures and Tables

**Table 1:** Baseline characteristics of ME/CFS patients with a %CBF reduction in the normal range of healthy controls during a tilt test, patients with an abnormal %CBF reduction, and healthy controls.

	ME/CFS with Abnormal %CBF Reduction (n = 488)	ME/CFS with Normal %CBF Reduction (n = 46)	Healthy Controls (n = 49)	*p*-Value: Chi-Square (*) One-Way Welch ANOVA with Post Hoc (∞); Kruskal–Wallis with Post-Hoc (#) Mann–Whitney U (##)
Male/female	70/418 (14/86%)	19/27 (41/59%)	11/38 (22/78%)	* *p* < 0.001
Age (years)	41 (12)	44 (11)	39 (15)	∞ F (2, 580) = 1.540 *p* = 0.215
Height (cm)	171 (8)	174 (10)	173 (7)	∞ F (2, 580) = 3.377 *p* = 0.035
Weight (kg)	70 (62–81)	74 (62–85)	73 (63–82)	# X^2^ (2) = 1.788 *p* = 0.409
BMI (kg/m^2^)	23.8 (21.1–27.7)	24.2 (21.2–27.4)	24.3 (21.3–27.6)	# X^2^ (2) = 0.089 *p* = 0.956
BSA (m^2^)	1.84 (0.20)	1.88 (0.21)	1.87 (0.19)	∞ F (2, 580) = 1.592 *p* = 0.204
Disease duration (years)	12 (6–20)	13 (7–21)	NA	## *p* = 0.76
Disease severity ©	141/263/84 (29/54/17%)	35/10/1 (76/22/2%)	NA	* *p* < 0.001

* Chi-square 2 × 2 or 2 × 3 analysis; ∞: one-way Welch ANOVA with Tukey/Games–Howell post hoc; ##: median (IQR) Mann–Whitney U; #: Kruskal–Wallis test with Dunn post hoc; BMI: body mass index: BSA: body surface area (formula duBois); CBF: cerebral blood flow; ME/CFS: myalgic encephalomyelitis/chronic fatigue syndrome; ©: disease severity presented as the % with mild/moderate/severe disease according to the ICC [29]. A *p*-value of <0.01 is considered statistically significant.

**Table 2:** Tilt test data of ME/CFS patients with a %CBF reduction in the normal range of healthy controls during a tilt test, patients with an abnormal %CBF reduction, and healthy controls.

	ME/CFS with Abnormal %CBF Reduction (n = 488)	ME/CFS with Normal %CBF Reduction (n = 46)	Healthy Controls (n = 49)	*p*-Value: Chi-Square (*) One-Way Welch ANOVA with Post Hoc (∞); Kruskal–Wallis with post Hoc (^#^)
HR supine (bpm)	69 (11)	67 (11)	62 (9)	∞ F (2, 580) = 10.814 *p* < 0.001 1 vs. 3: *p* < 0.001
HR end-tilt (bpm)	85 (14)	82 (11)	79 (15)	∞ F (2, 580) = 5.480 *p* = 0.004 1 vs. 3: *p* = 0.006
HR increase (bpm)	16 (8)	15 (7)	17 (9)	∞ F (2, 580) = 0.947 *p* = 0.469
CO supine (L/min)	4.64 (4.10–5.30)	4.40 (3.91–4.98)	4.27 (3.74–4.70)	^#^ X^2^ (2) = 17.601 *p* < 0.001 1 vs. 3: *p* < 0.001 ^®^
CBF supine (mL/min)	617 (100)	599 (97)	620 (84)	∞ F (2, 580) = 0.769 *p* = 0.464
CBF end-tilt (mL/min)	440 (78)	567 (91)	580 (82)	∞ F (2, 580) = 112.646 *p* < 0.001 1 vs. 2: *p* < 0.001; 1 vs. 3: *p* < 0.001
%CBF reduction end-tilt	−29 (6)	−5 (2)	−6 (3)	∞ F (2, 580) = 750.409 *p* < 0.001 1 vs. 2: *p* < 0.001; 1 vs. 3: *p* < 0.001
P_ET_CO_2_ reduction (mmHg)	−8 (4) (n = 440)	−2 (3) (n = 21)	−2 (2) (n = 48)	∞ F (2, 506) = 64.397 *p* < 0.001 1 vs. 2: *p* < 0.001; 1 vs. 2: *p* < 0.001 1 vs. 3: *p* < 0.001
%pat P_ET_CO_2_ < 30/≥30 mmHg	189/251 (43/57%)	0/21 (0/100%)	1/47 (2/98%)	* *p* < 0.001
SBP supine (mmHg)	137 (18}	138 (15)	135 (16)	∞ F (2, 580) = 0.299 *p* = 0.741
SBP end-tilt (mmHg)	134 (18)	135 (15)	127 (15)	∞ F (2, 580) = 4.037 *p* = 0.018 post hoc: ns
DBP supine (mmHg)	79 (73–86)	80 (72–85)	79 (74–83)	^#^ X^2^ (2) = 0.432 *p* = 0.806
DBP end-tilt (mmHg)	85 (78–93)	86 (79–93)	80 (76–88)	^#^ X^2^ (2) =9.373 *p* = 0.009 1 vs. 3: *p* = 0.008 ^®^
MAP supine (mmHg)	102 (94–110)	104 (93–110)	102 (95–107)	^#^ X^2^ (2) = 0.655 *p* = 0.721
MAP end-tilt (mmHg)	103 (95–113)	105 (95–111)	96 (90–107)	^#^ X^2^ (2) = 12.612 *p* = 0.002 1 vs. 3: *p* = 0.008 ^®^
%MAP increase end-tilt	2 (−3–7)	2 (−2–6)	−1 (−11–4)	^#^ X^2^ (2) = 11.527 *p* = 0.003 1 vs. 3: *p* = 0.002 ^®^
Tilt duration (min)	13 (7–13)	25 (19–28)	25 (23–28)	¶ X^2^ (2) = 124.092 *p* < 0.001 1 vs. 2: *p* = 0.000 1 vs. 3: *p* = 0.000 ^®^

BSA: body surface area (formula duBois); CBF: cerebral blood flow; CO: cardiac output; DBP: diastolic blood pressure; HR: heart rate; MAP: mean arterial pressure; ME/CFS: myalgic encephalomyelitis/chronic fatigue syndrome; P_ET_CO_2:_ end-tidal CO_2_ pressure; SBP: systolic blood pressure; * Chi-square 2 × 2 or 2 × 3 analysis; ∞: one-way Welch ANOVA with Tukey/Games–Howell post hoc; ^#^: median (IQR) Kruskal–Wallis test with Dunn post hoc ^®^: Bonferroni correction for multiple tests. ¶: tilt duration was calculated from the onset of the tilt to the last VTI acquisition. Ns: not statistically signifant. A *p*-value of <0.01 is considered statistically significant.

**Table 3:** Analysis of the possible relationship between percentage cerebral blood flow reduction during the tilt test and clinical and hemodynamic variables in ME/CFS patients with a normal heart rate and blood pressure response during the tilt and an abnormal CBF reduction.

Variable	N = 440 ^®^		
Gender: M	%CBF = −0.306 × F − 28.762	R^2^ = 0.000	*p* = 0.686
Age (years)	%CBF = 0.042 × age − 30.536	R^2^ = 0.008	*p* = 0.054
Disease duration (years)	%CBF = 0.008 × DisDur − 28.697	R^2^ = 0.000	*p* = 0.756
Disease severity: mild ©	%CBF = −1.516 × moderate − 2.537 × severe − 27.492	R^2^ = 0.024	*p* = 0.005
Length (cm)	%CBF = −0.004 × length − 28.109	R^2^ = 0.000	*p* = 0.897
Weight (kg)	%CBF = 0.022 × weight − 30.380	R^2^ = 0.004	*p* = 0.188
HR increase (bpm)	%CBF = −0.051 × HR increase − 29.606	R^2^ = 0.006	*p* = 0.12
%CO reduction	%CBF = 0.974 × %CO reduction − 0.908	R^2^ = 0.778	*p* < 0.001
P_ET_CO_2_ reduction (mmHg)	%CBF = 0.324 × P_ET_CO_2_ reduction − 26.328	R^2^ = 0.062	*p* < 0.001
End-tilt P_ET_CO_2_ < 30 mmHg	%CBF = −2.361 × P_ET_CO_2_E-T − 27.792	R^2^ = 0.045	*p* < 0.001
%MAP increase (mmHg)	%CBF = −0.036 × %MAP increase − 28.709	R^2^ = 0.003	*p* = 0.250
Tilt duration (min) ¶	%CBF = 0.092 × TiltDur − 30.068	R^2^ = 0.012	*p* = 0.020

%CBF: percentage cerebral blood flow reduction during tilt compared to supine CBF; CO: cardiac output; DisDur: disease duration; F: female; HR: heart rate; M: male; MAP: mean arterial pressure; ME/CFS: myalgic encephalomyelitis/chronic fatigue syndrome; P_ET_CO_2_: end-tidal CO_2_ pressure; P_ET_CO_2_E-T: end-tilt P_ET_CO_2_; TiltDur: tilt duration; ©: disease severity according to the ICC [29], ¶: tilt duration was calculated from the onset of the tilt to the last VTI acquisition, ^®^: subset of patients with a complete P_ET_CO_2_ set (90% of all patients).

**Table 4:** Multiple logistic regression in ME/CFS patients with an abnormal percentage cerebral blood flow reduction to predict the percentage cerebral blood flow reduction during the tilt by other variables.

	B	t	*p* Value
(Constant)	−0.048	−0.048	*p* = 0.962
Age (years)	0.015	1.437	*p* = 0.151
Dummy moderate *	−0.307	−1.003	*p* = 0.316
Dummy severe *	−0.663	−1.731	*p* = 0.084
%CO reduction	0.959	37.399	*p* < 0.001
P_ET_CO_2_ reduction (mmHg)	0.100	2.555	*p* = 0.011
End-tilt P_ET_CO_2_ < 30 mmHg	−0.083	−0.265	*p* = 0.791
Tilt duration (min) ¶	−0.063	−3.008	*p* = 0.003

%CO: percentage cardiac output reduction during the tilt compared to supine; ME/CFS: myalgic encephalomyelitis/chronic fatigue syndrome; P_ET_CO_2_: end-tidal CO_2_ pressure. * Disease severity according to the ICC [29], ¶: tilt duration was calculated from the onset of the tilt to the last VTI acquisition.

## Data Availability

Not applicable.

## Supplementary Materials

The following supporting information can be downloaded at: https://www.mdpi.com/article/10.3390/healthcare12242566/s1, Figure S1: Flow diagram of ME/CFS patients and healthy controls.
