# Supplementary material for: The Cardiac Output–Cerebral Blood Flow Relationship Is Abnormal in Most Myalgic Encephalomyelitis/Chronic Fatigue Syndrome Patients with a Normal Heart Rate and Blood Pressure Response During a Tilt Test
Source: Healthcare (Basel). 2024 Dec 20;12(24):2566. doi: 10.3390/healthcare12242566 (PMC11675211; doi:10.3390/healthcare12242566)
Supplement: Supplementary file 1 [file healthcare-12-02566-s001.zip › healthcare-3283848-supplementary.pdf]

Supplementary Materials

The supplementary Figure S1 shows the flow diagram of ME/CFS patient and healthy control inclusion.

Supplementary Figure S1. Flow diagram of ME/CFS patients and healthy controls.

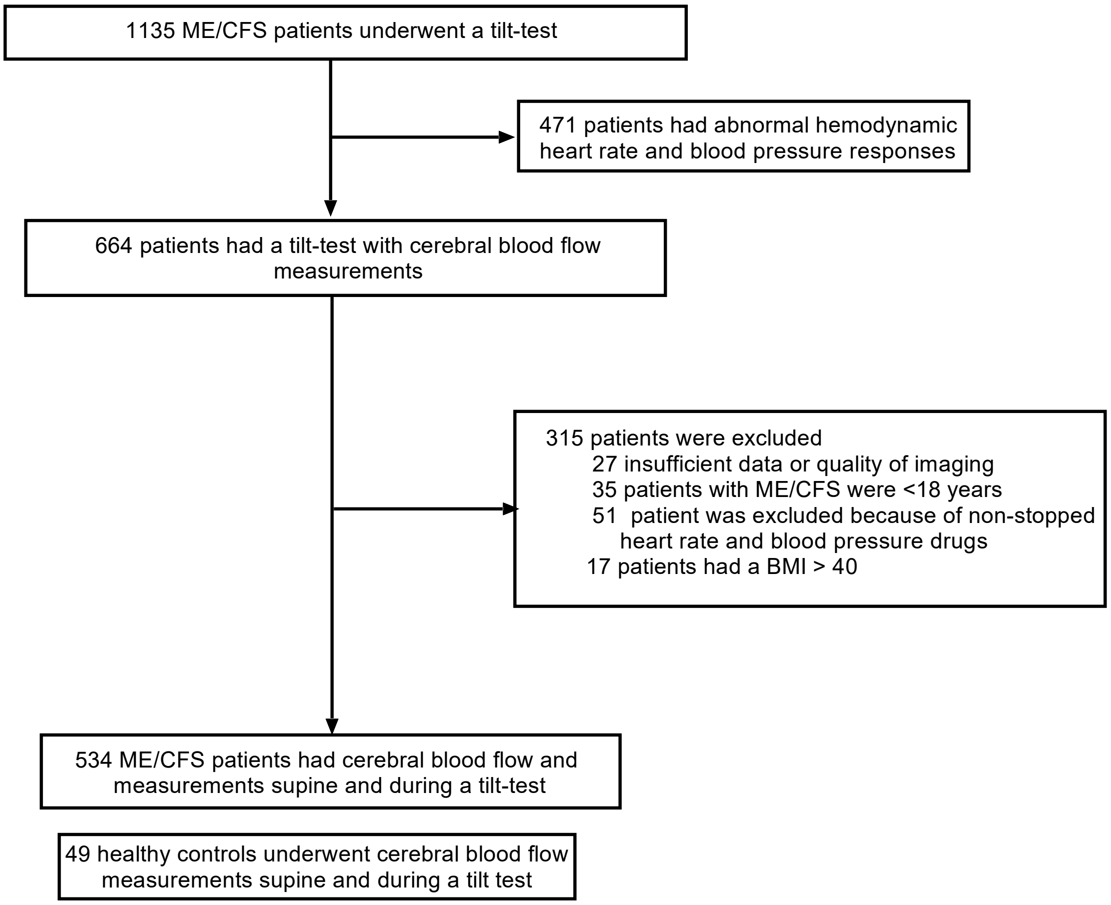

Legend supplementary Figure S1: ME/CFS: myalgic encephalomyelitis/chronic fatigue syndrome; norm: normal.
